# Protein profiling of forehead epidermal corneocytes distinguishes frontal fibrosing from androgenetic alopecia

**DOI:** 10.1371/journal.pone.0283619

**Published:** 2023-03-31

**Authors:** Noreen Karim, Paradi Mirmirani, Blythe P. Durbin-Johnson, David M. Rocke, Michelle Salemi, Brett S. Phinney, Robert H. Rice

**Affiliations:** 1 Department of Environmental Toxicology, University of California, Davis, California, United States of America; 2 Department of Dermatology, The Permanente Medical Group, Vallejo, California, United States of America; 3 Department of Public Health Sciences, Division of Biostatistics, Clinical and Translational Science Center Biostatistics Core, University of California, Davis, California, United States of America; 4 Proteomics Core Facility, University of California, Davis, California, United States of America; INSERM, FRANCE

## Abstract

Protein profiling offers an effective approach to characterizing how far epidermis departs from normal in disease states. The present pilot investigation tested the hypothesis that protein expression in epidermal corneocytes is perturbed in the forehead of subjects exhibiting frontal fibrosing alopecia. To this end, samples were collected by tape stripping from subjects diagnosed with this condition and compared to those from asymptomatic control subjects and from those exhibiting androgenetic alopecia. Unlike the latter, which exhibited only 3 proteins significantly different from controls in expression level, forehead samples from frontal fibrosing alopecia subjects displayed 72 proteins significantly different from controls, nearly two-thirds having lower expression. The results demonstrate frontal fibrosing alopecia exhibits altered corneocyte protein expression in epidermis beyond the scalp, indicative of a systemic condition. They also provide a basis for quantitative measures of departure from normal by assaying forehead epidermis, useful in monitoring response to treatment while avoiding invasive biopsy.

## Introduction

Proteomic profiling can provide considerable information about the differentiated or pathological state of corneocytes and complex structures comprised of them [1]. The major proteins of epidermal stratum corneum [2,3], hair shaft [4,5] and nail plate [6] have all been identified by this means. Substantial differences in profile are evident in epidermal corneocytes in disease states as a result of genetic manipulation in the mouse [7,8] or of defective (mutated) alleles in the human population [9,10]. In such work, proteomic analysis revealed the great impact on the profile even of single amino acid changes in mutated proteins of interest.

In men and women who develop hair thinning due to androgenetic alopecia (AGA, pattern hair loss), there is a complex interplay between androgens and multiple susceptibility genes that leads to miniaturization of the hair shaft. The observed wide variation in individual sensitivity and severity reinforces the likely multifactorial nature of the condition. However, recent genome wide association studies have confirmed that AGA has a genetic component [11]. The association of AGA with numerous pathological conditions may be rationalized by identifying common responsible signaling pathways among the many that have been implicated [12,13]. Another factor that likely contributes to miniaturization of hair follicles in AGA is the observed mild perifollicular inflammation, particularly in the lower infundibulum near stem cells of the bulge, and fibrosis preventing descent of transit amplifying cells to form the anagen hair bulb [14].

Frontal fibrosing alopecia (FFA) is a distinct and increasingly commonly diagnosed [15] form of cicatricial alopecia with permanent hair loss affecting the frontal hairline in a bandlike pattern and often accompanied by loss of eyebrows [16]. Additional clinical findings can include facial papules, cutaneous pigmentary changes, and loss of eyelashes and body hair, indicating that the condition may not be limited to the scalp [17]. Histological features (including severe perifollicular inflammation and concentric lamellar fibroplasia) are difficult to distinguish from lichen planopilaris [18]. Most frequently found in postmenopausal females, it can occur before menopause as well as infrequently in males. A genetic predisposition seems likely from analysis of familial relationships [19,20], a possibility supported by identification of several genomic loci, including an HLA allele, associated with it by genome wide studies [21]. Lichen planopilaris and FFA have been hypothesized to result from damage to hair follicle stem cells due to an autoimmune inflammatory response upon collapse of immune privilege [22,23]. This collapse may be triggered by a loss of normal interferon (IFN)-γ and peroxisome proliferator-activated receptor (PPAR)-γ-mediated signaling and homeostasis in the folliculosebaceous unit [24]. Factors suggested to contribute to the occurrence of FFA include alterations in hormone levels or hair follicle microbiomes, defective mitochondrial lipid metabolism, neurogenic inflammation, perturbed levels of aryl hydrocarbon receptor pathway components and environmental factors such as cutaneous allergens and fragrances from personal care products [23,25].

The present pilot work investigates whether FFA affects the corneocyte protein profile in the interfollicular epidermis beyond the scalp. AGA samples were analyzed in parallel, since a systemic effect of this condition has not been reported. The results revealed little difference between protein profiles of forehead epidermis from AGA and non-symptomatic control subjects, but a dramatic difference between these and FFA forehead samples. This finding provides clear evidence of epidermal perturbation beyond the scalp in FFA, consistent with the observed systemic influence.

## Methods

### Subject recruitment and metadata

Samples were collected from 5 patients (4 female, 1 male) with FFA (ages 70.4 ± 2.5), all of whom were diagnosed by the same investigator (PM), 5 male subjects with AGA (ages 51.6 ± 15.5) and 12 control individuals without hair loss, 6 males (ages 64.8 ± 7.4) and 6 females (ages 61.3 ± 6.6) (University of California, Davis). The hair loss in the AGA subjects was Norwood class 5a, while that in FFA subjects was pattern II [26], with FFA Severity Scores of 15.1 ± 3.1 and each subject having extensive disease with ongoing inflammation. Samples from shaved normal scalp, of uncertain suitability, were difficult to obtain and were not collected. All the FFA subjects were under treatment, one with oral pioglitazone (primarily a PPARγ agonist) and 4 with topical corticosteroids, of which one was co-treated with oral pioglitazone, one with oral plaquenil (suppressor of Toll-like receptors) and two with oral dutasteride (blocking conversion of testosterone to dihydrotestosterone by 5α-reductase), none of which reverse or completely prevent disease progression in most cases [26]. From each subject, samples of 5 tape circles (CuDerm D-squame adhesive circles, 2.2 cm diameter) were collected from the forehead at equidistant sites roughly covering the area. Similarly, 5 tape circle samples were collected from the scalp, covering the affected area without hair, from each individual with FFA and AGA. The study was conducted in accordance with the protocols and procedures approved by the Institutional Review Board of the University of California, Davis (IRB#217868), and written informed consent was obtained from each subject before sampling.

### Sample processing

As previously described [3], tape circles with attached corneocytes were held in 2% sodium dodecyl sulfate– 0.1M sodium phosphate buffer (pH 7.8) overnight, allowing the keratinocytes to elute from the circles and settle at the bottoms of the tubes. The keratinocytes from each sample were transferred to a clean microfuge tube followed by centrifugation. The supernatant was discarded, and the pellet was washed twice by re-suspension in 2% sodium dodecanoate– 0.05 M NH_4_HCO_3_ followed by centrifugation. The resulting pellet was then re-suspended in 0.4 mL of 2% sodium dodecanoate– 0.0.05 M NH_4_HCO_3_. After addition of dithioerythritol to 50 mM, samples were incubated at 95°C for 15 min followed by magnetic stirring for 45 min at room temperature. Sulfhydryls were alkylated with iodoacetamide (100 mM) with stirring for 45 min in the dark. Sodium dodecanoate was removed by extracting three times with 700 μL of ethyl acetate after adjusting the pH to ~3 with trifluoroacetic acid. The aqueous layer was readjusted to pH ~8.5 with 2.5 μL of concentrated ammonium hydroxide and 20 μL of 1M NH_4_HCO_3_ before addition of 20 μg of reductively methylated trypsin for protein digestion. Digestion was continued for 3 days with daily additions of 20 μg of methylated bovine trypsin [27]. The samples were clarified by centrifugation and stored frozen at -80°C until analysis. The digests were quantitated by fluorescent peptide analysis and, on that basis, 600 ng of peptide material were analyzed by mass spectrometry.

### Mass spectrometry and generation of weighted spectral counts

Randomized protein digests were analyzed by LC-MS/MS using a Thermo Scientific Dionex UltiMate 3000 RSLC system with a PepSep (Denmark) ReproSil 8 cm 150 μm diameter C18 column with 1.5 μm particle size (120 Å pores) at 40°C. Separation was performed with a flow rate of 0.5 μl/min for 60 min using mobile phases (a) 0.1% formic acid in water and (b) 80% acetonitrile/0.1% formic acid. The eluted peptides were directly applied to an Orbitrap Exploris 480 mass spectrometer (Thermo Fisher Scientific, Bremen, Germany) using a spray voltage of 1.8 kV and heated capillary temperature set to 275°C. A full MS resolution of 60,000 at m/z 200% and full MS automatic gain control target of 300% were used with a mass range of 350–1500. The automatic gain control target value for fragment spectra was set to 200% with a resolution of 15,000. Isolation width and normalized collision energy were set to 1.5 m/z and 30%, respectively. The data were searched (one missed tryptic cleavage was allowed) against the HumanFR_crap05292020_rev database (149661 entries) with appended identical but reversed (decoy) peptides and common human contaminants using X! Tandem Alanine (2017.2.1.4) essentially as previously described [3]. The search was performed with the fragment ion mass tolerance of 20 ppm and parent ion tolerance of 20 ppm initially with subsequent screening at 5 ppm, cysteine carbamidomethylation as fixed modification, and with N-terminal ammonia loss, N-terminal glutamate or glutamine pyrolysis (Glu/Gln→pyroGlu), asparagine and glutamine deamidation, and oxidation or deoxidation of methionine and tryptophan as variable modifications. Proteins with shared peptides were grouped using Scaffold software (version 5.2.1). Peptide identifications found to be established at >95% probability by Scaffold LFDR algorithm were accepted, while the protein identifications were accepted if corroborated at >99% probability with at least two identified peptides. This stringent criterion reduced the peptide decoy FDR to <0.1% and protein decoy FDR to 1.2%. Proteins that could not be differentiated by MS/MS analysis solely, due to presence of shared peptides, were clustered to satisfy parsimony principles. As previously observed [4], numerous semi-tryptic peptides were obtained in the digests (≈40% of the total). They showed the same protein identification specificity in this work as full tryptic peptides judging by distribution between given proteins and clusters and by estimated false discovery rate. The weighted spectral count values for the proteins were compared to the spectral counts of exclusive peptides (peptides belonging to only one protein), and the proteins with numerous weighted counts but no or few exclusive peptides were omitted from the analysis. As listed in S1 Table, the 277 proteins with the highest weighted spectral counts (each detected with more than an average of 1 spectral count per sample) were then submitted for statistical analysis.

### Label free quantitation

Since spectral counts are not suitable for estimating relative amounts of different proteins, label free quantitation was employed for this purpose. The MS data for all the samples were searched against a validated UNIPROT human reference proteome (uniport-proteome_UP000005640) using PEAKS Studio 10.6 (Bioinformatics Solutions Inc., Waterloo, ON, Canada) with settings as previously described [27]. The proteomes obtained were used to quantitate the abundances of proteins in each study group based on the area values of their top 3 peptides. Normalized values for the different categories are given in S2 Table. The raw data, Scaffold file and PEAKS Studio output files are available in the MASSive Proteomics repository (massive.ucsd.edu/#MSV000090141) and ProteomeExchange (http://www.proteomexchange.org/#PXD036085).

### Statistical analysis

Differential protein expression analyses were conducted using weighted spectral counts [28,29] obtained from the Scaffold output for better sensitivity at low protein abundance [30] and analyzed by the limma-voom Bioconductor pipeline [31], which was originally developed for RNA sequencing data (limma version 3.44.1, edgeR version 3.30.1). Normalization factors were calculated using TMM [32]. The model used in limma included effects for location, condition (or sex), their interaction and batch. Standard errors of log fold changes were adjusted for within-subject correlations. Analyses were conducted using R version 4.0.0 Patched (2020-05-18 r78487). Differences were considered significant when p<0.05 was observed after correction for multiple testing [33]. Multidimensional scaling (MDS) plots were conducted using the function plotMDS in edgeR and used classical multidimensional scaling [34].

## Results

### Asymptomatic control samples

Present work focused on alterations in FFA and AGA protein profiles of corneocytes in the epidermis to determine the degree of divergence from normal. Forehead samples from asymptomatic control individuals were compared first. The present analysis showed little difference between males and females in forehead epidermal profiles. The level of only one protein was found to be different, with the plakophilin-3 level in samples from males being on average 5–6 times that in samples from females (Table 1 and S3A Table). To provide a basis in subsequent comparisons for judging the relative prevalence of the identified proteins, data from asymptomatic control forehead samples were analyzed by label free quantitation. As seen in Table 2 (and S2 Table), 16 of the 33 most prevalent identified proteins (each estimated ≥0.1% of total protein) were keratins. As is well known for cells of the stratum corneum, keratins comprised the large majority of the proteome in the forehead samples (90%), similar to the previously observed content in forearm corneocytes, also sampled by tape stripping [10].

**Table 1:** Pairwise comparisons of protein expression level.

Category	Diff
**Forehead**	
Male vs Female	1
AGA vs Control	3
FFA vs Control	72
**FFA vs AGA**	
Forehead	27
Scalp	22
**Scalp vs Forehead**	
AGA	8
FFA	16

**Table 2:** Relative amounts of proteins identified in asymptomatic forehead stratum corneum by tape stripping.

Protein	%
KRT10	41.8
KRT1	26.1
KRT2	14.3
KRT5	1.9
KPRP	1.9
***S100A9***	***0*.*9***
KRT14	0.8
XP32	0.7
***KRT85***	***0*.*6***
***S100A8***	***0*.*6***
***KRT31***	***0*.*6***
KRT9	0.5
***KRT16***	***0*.*4***
DSC1	0.4
KRT17	0.3
LOR	0.3
***KRT77***	***0*.*3***
KRT78	0.3
DSG1	0.3
DSP	0.3
***KRT86***	***0*.*3***
KRT6A	0.3
***KRTAP3-1***	***0*.*2***
JUP	0.2
FLG2	0.2
CNFN	0.2
KRT33B	0.2
KRT33A	0.1
ANXA2	0.1
TGM1	0.1
ALOX12B	0.1
***TXN***	***0*.*1***
FLG	0.1

Samples from forehead were used to compare profiles from (a) asymptomatic male versus female subjects, (b) androgenetic (AGA) versus asymptomatic control subjects and (c) frontal fibrosing alopecia (FFA) versus control subjects. Samples from forehead and scalp were used to compare profiles in FFA versus AGA subjects. Comparisons were also made between scalp and forehead profiles of AGA and FFA subjects. Diff = numbers of proteins significantly different in expression level; lists of these proteins and the fold difference in level are provided in S3 Table.

Estimates of protein amounts derived from label free quantitation were normalized to 100% for the 263 proteins identified. Those with estimated levels of at least 0.1% are illustrated. Of these 33 most prevalent proteins, 9 (bold italics) were seen to differ in level between FFA and control forehead samples. The full list of proteins is given in S2 Table along with parallel quantitations using AGA and FFA forehead samples.

### FFA and AGA versus control samples

**Fig 1:**
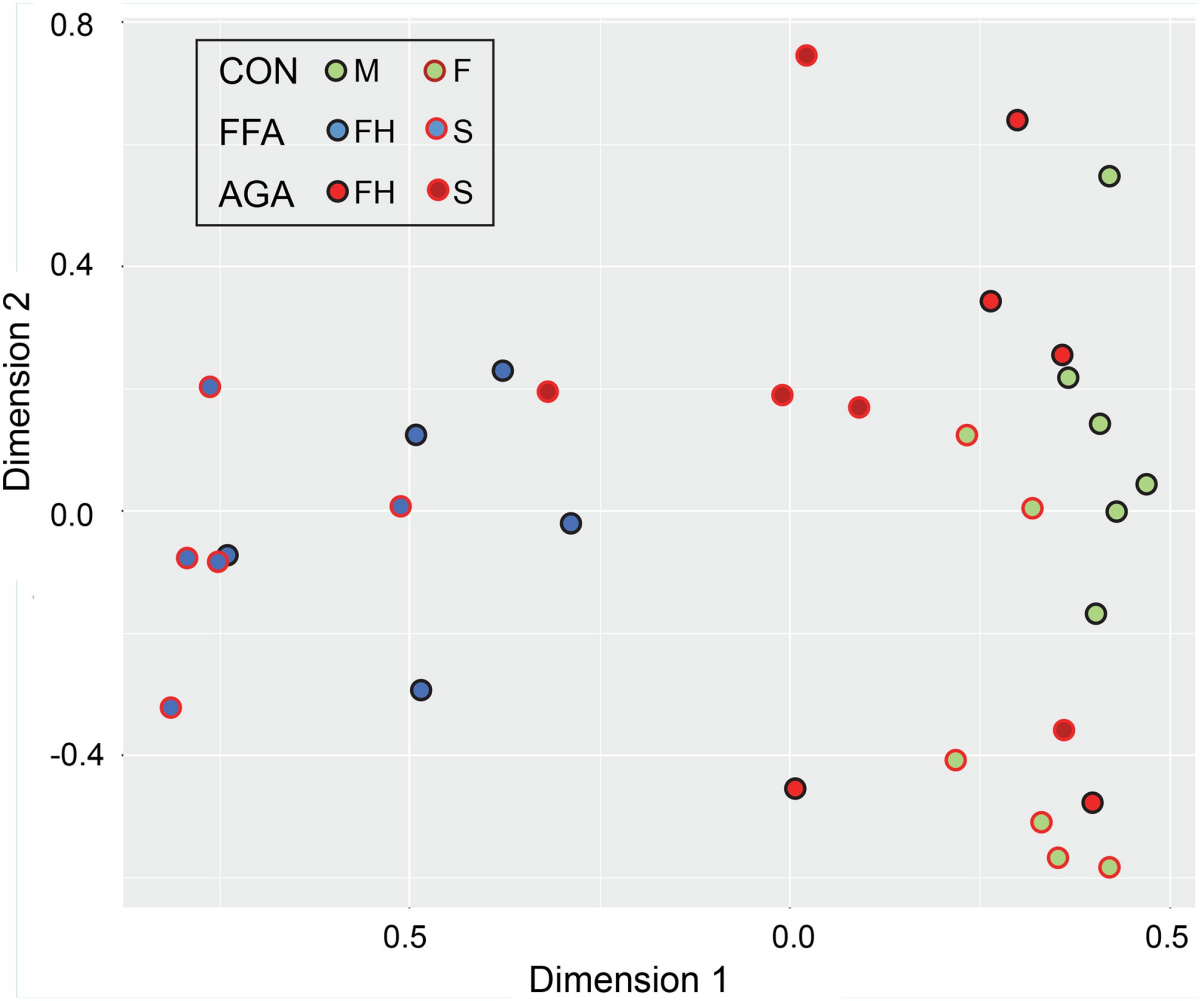
Multidimensional plot of data from forehead samples. Although definite interindividual differences among subjects are seen, particularly noticeable in the AGA cohort, a clear separation is evident between FFA and control cohorts.

The protein profiles of samples from the FFA, AGA and asymptomatic control subjects were analyzed by 2-way comparisons based on the weighted spectral counts from S1 Table and using the statistical model described above. Comparisons included samples collected from the scalp as well as forehead of AGA and FFA subjects. Results of pairwise comparisons are summarized in Table 1. A multidimensional plot comparing the forehead protein profiles to each other showed the FFA samples relatively close to each other and well separated from the control samples, while the AGA samples, mostly well separated from FFA samples, were overall much closer to the controls (Fig 1).

**Fig 2:**
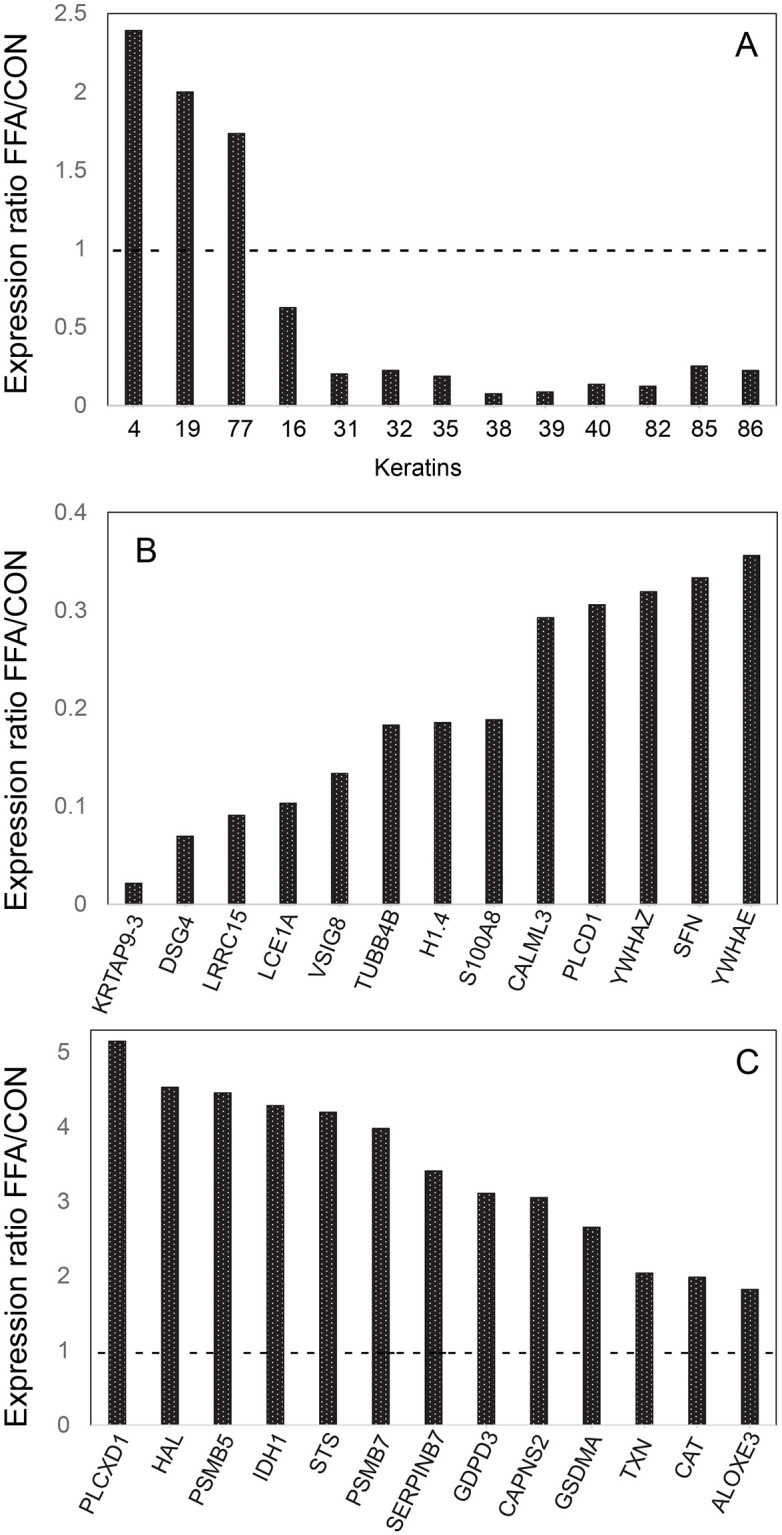
Ratios of expression levels of proteins found significantly different in FFA compared to asymptomatic control forehead samples. Illustrated are (A) 13 keratins that were expressed at significantly different levels in FFA samples, (B) 13 representative proteins found at lower levels in FFA samples, and (C) 13 representative proteins found at higher levels in FFA samples.

Forehead profiles from FFA subjects differed dramatically from the controls, where 72 proteins showed significant differences, 25 at higher and 47 at lower levels than in control samples (S3 Table). Among these, 13 keratins were conspicuous, with 3 expressed at higher and 10 at lower levels (Fig 2A). Of the latter, the 9 lowest are classified as “hair” keratins and the others as “epithelial” [35]. Fig 2B and 2C each show relative levels of 13 representative other proteins expressed at lower and higher levels, respectively. Among those shown in Fig 2B, members of 3 protein families were suppressed. In addition to keratin associated protein 9–3 (KRTAP9-3), KRTAPs 9–9, 2–3, 13–2, 16–1, 10–10 and 3–1 were expressed at 2%, 6%, 12%, 15% and 17% of control levels, respectively (S3 Table). Similarly, in addition to S100A8, S100s A3, A2, A7 and A9 were expressed at 20%, 23%, 39% and 58% of levels in the controls. Finally, members of the 14-3-3 adaptor family YWHAZ, SFN and YWHAE were expressed at ≈35% of control samples (Fig 2B).

From the list of differentially expressed proteins identified in FFA versus asymptomatic control forehead samples, Ingenuity Pathway Analysis (IPA) software was used to find possibly perturbed signaling pathways. Only two such pathways were identified, both showing high probability of being suppressed (Z scores ≤-2) (S1 Fig). Both contained phospholipase C delta 1 (PLCD1) and the three 14-3-3 adaptor proteins SFN, YWHAE, YWHAZ (Fig 2B). The altered expression of these and certain other proteins are consistent with the phenotype of FFA (see Discussion).

### AGA versus control and scalp samples

**Fig 3:**
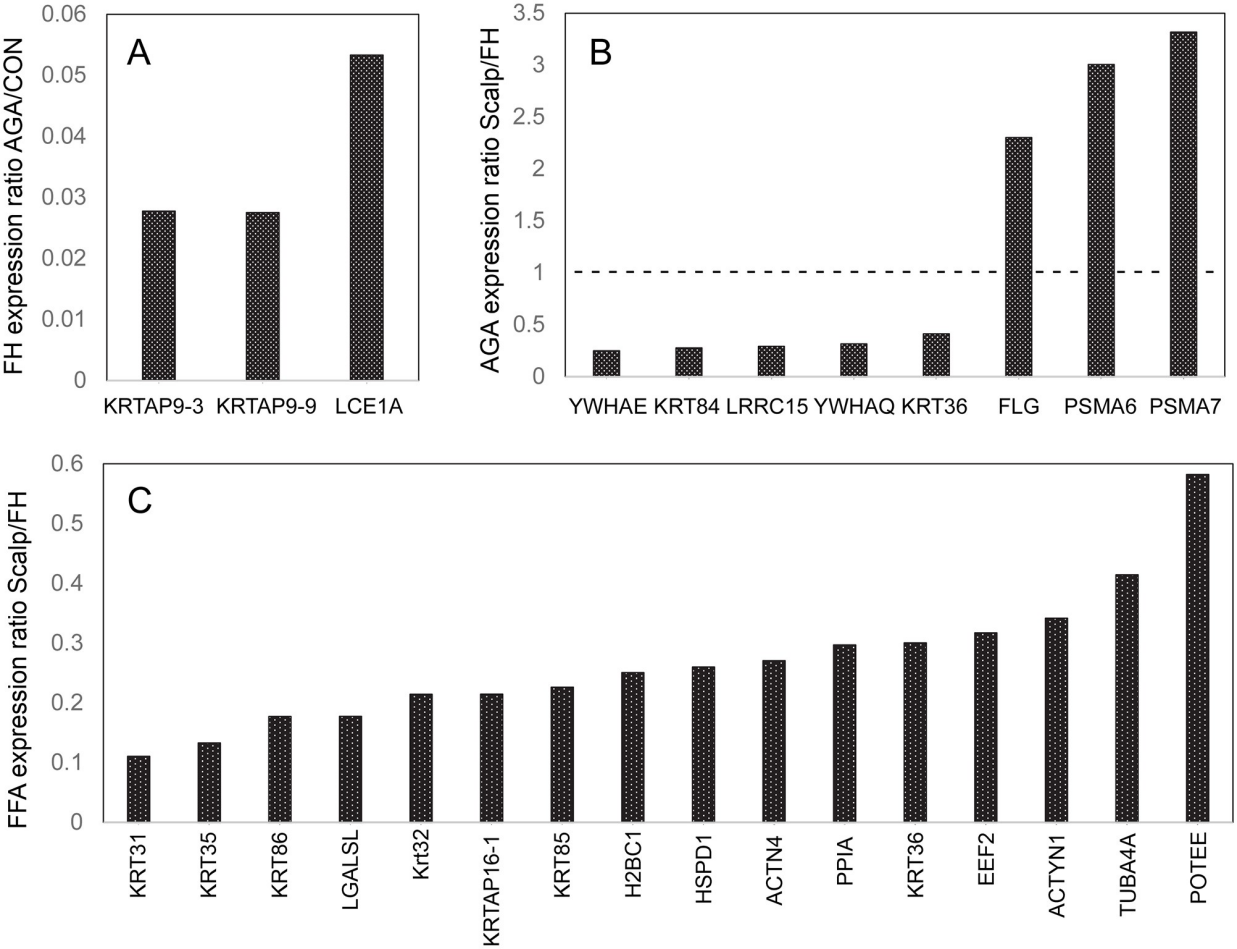
Ratios of expression levels in AGA and FFA samples compared to control or scalp compared to forehead. Shown are ratios of expression level of (A) AGA to control (CON) from forehead samples, (B) scalp to forehead (FH) from AGA samples and (C) scalp to forehead from FFA samples. In (A), protein expression levels illustrated were lower in AGA than control, and in (B) and (C) most of the protein levels illustrated were lower in scalp than in forehead.

Forehead protein profiles from subjects with AGA differed little from those from asymptomatic control individuals. Levels of only three proteins were significantly different, all much lower than in the control (Fig 3A). These were also seen to be suppressed to nearly the same extents in the FFA forehead samples. Since the protein profile from forehead of FFA subjects was so different from controls, one would expect the FFA profile to differ nearly as much from the forehead AGA profile. Indeed, comparison of expression levels in FFA versus AGA in the forehead revealed a total of 27 proteins that were significantly different. Although substantial, this number was considerably fewer than the total of 72 differences between FFA and control samples. This contrast appeared due to perturbation of the levels in AGA samples that were not divergent enough to be found statistically significant from the control values but were sufficiently different (in the same direction as in FFA) to reduce the degree of difference (and significance) between FFA and AGA levels (S2 Fig).

The question arose whether the results from the forehead samples reflect similar perturbations in the scalp epidermis. Comparison of AGA forehead samples with AGA scalp samples displayed only 8 differences (Fig 3B). Analogous to the FFA versus control forehead comparison (Fig 2), AGA scalp samples were markedly higher than those from AGA forehead in the proteasomal proteolytic subunits PSMA6 and PSMA7 and considerably lower in the 14-3-3 members YWHAE and YWHAQ and the two hair keratins K36 and K84. Despite the lack of available asymptomatic subjects with the FFA scalp treatments to use as controls, we compared the samples collected from the forehead and scalp of FFA subjects to each other. The comparison showed 16 significant protein differences (Fig 3C), much fewer than the 72 differences between samples from FFA forehead and control forehead. Strikingly, the scalp samples were even lower in the 6 hair keratins 31, 32, 35, 36, 85 and 86. While this comparison is only suggestive, since the influence of the topical FFA scalp treatments was not studied, the scalp profile thus appeared similar to that in FFA forehead samples.

## Discussion

FFA is characterized by an inflammatory attack on the hair follicles of the scalp, resulting in a receding hairline. The general finding of accompanying hair loss in the eyebrows and frequently elsewhere, including the extremities, has pointed to a more systemic immunologic phenomenon in men and women [36,37]. Present results confirm the hypothesis that effects are detectable beyond the hair follicles, evident in the stratum corneum of forehead, presumably resulting from perturbation of signaling in the spinous cells. Compared to the profound effects in FFA samples (72 proteins different from control), the disease process in AGA showed a considerably smaller effect on interfollicular corneocytes of the forehead (3 proteins different from control).

Epidermal keratinocytes are well known to participate in inflammatory phenomena by secreting cytokines and responding to those secreted in their vicinity. If perturbations of keratinocyte protein expression levels could be attributed to specific cytokine signaling pathways, protein profiling might help elucidate sources of the inflammation. A number of cytokines are known to suppress keratin levels, especially in combination [38]. Suppression of major keratins K1 and K10 as well as filaggrin occurs in cultured human epidermal keratinocytes in response to several interleukins [39,40], but these proteins were not significantly altered in the current work. Present results did not reveal significant effects on major corneocyte proteins found to comprise ≥ 1% of the proteome (including KRTs 1, 2, 5, 10, 14). Thus, the minor proteins identified here are considerably more useful in evaluating the degree of inflammatory effect. These include keratins ordinarily associated with hair shafts. In AGA, terminal hairs are targeted for miniaturization while, in contrast, vellus hairs in FFA are lost despite the persistence of isolated terminal hairs [41]. The observed loss of hair keratins shown in this work is consistent with targeting of vellus hairs in FFA.

Signaling pathway analysis highlighted possible contributions of reduced expression of PLCD1 and three 14-3-3 adaptor proteins to the observed pathological state. Mice with nonfunctional PLCD1, normally a negative regulator of proinflammatory cytokine production in macrophages [42], display alopecia and an inflammatory phenotype in the skin [43,44]. In addition, loss of PLCD1, downstream in the Foxn1 signaling pathway and lacking in the nude mouse, results in low levels of the 6 keratins examined (Krt31-36) in the skin [45], similar to present observations in human forehead epidermis. Moreover, the low level of calmodulin like-3 protein (CALML3), which reflects the degree of keratinocyte differentiation independent of the proliferation state [46], is consistent with the low keratin levels in FFA samples.

The 14-3-3 adaptor family, containing 7 members with a high degree of sequence identity, stimulate protein interactions by binding to target proteins [47]. Thus, the observed reductions in their expression would be anticipated to attenuate the 14-3-3 signaling pathway. Since these adaptors bind to phosphoserine residues on their target proteins (>100 are known), which are phosphorylated by protein kinase A, reduced 14-3-3 protein expression would be expected to attenuate signaling by protein kinase A. Of these three adaptors, SFN (commonly called stratifin) is particularly noteworthy, since mice heterozygous for a frame shift mutation in the coding region exhibit repeated hair loss and regrowth [48,49]. Moreover, secreted by keratinocytes, stratifin stimulates collagenase activity in fibroblasts, reducing their collagen deposition [50]. Its loss is consistent with increased collagen deposition in FFA.

The present pilot data provide clues for further investigation of FFA pathogenesis. In addition to those implicated by the above pathways analysis, altered expression of several other proteins could be contributory factors. For example, an elevated level of ALOXE3 can increase lipid peroxidation and reactive oxygen production [51], consistent with the observed increase in thioredoxin (TXN) and catalase (CAT) levels. In addition, elevated GSDMA could raise the sensitivity of cells to pyroptosis, although molecular triggers for its activation are not known [52]. While increased immunoproteasomal subunit levels, stimulated by interferon gamma [53], were not observed, increases of the constitutive proteasomal proteolytic components PSMB5 and PSMB7 were seen, which could increase the generation of antigenic peptides presented on MHC-I molecules. On the other hand, often associated with increased inflammatory cytokine levels and inflammatory responses mediated by their binding to the receptor for advanced glycation end products and the Toll-like receptor 4, S100 calcium-binding proteins were lower in the FFA samples. However, their complex actions in cells [54] precludes clear expectations for the net effect.

In this pilot investigation, a preliminary evaluation of the scalp profiles of subjects with AGA and FFA was performed. Further evaluation of a larger FFA cohort and collection of treatment controls could help validate the findings, and present data could be the basis of useful hypothesis-driven investigation. While site specificity in callus profile has been demonstrated [2,3], the profile of bare scalp is likely quite similar to that of forehead. If this expectation is correct, then the data on FFA scalp samples can provide useful information, subject to verification. Measurements performed as in this pilot study could indicate the degree to which the treatments are effective in restoring the asymptomatic profile.

Present results show proteomic profiling of epidermal corneocytes provides quantitative information useful in characterizing departure from the normal state, although the basic causes of the observed departure remain unknown. Minimally invasive and painless, this approach can permit monitoring response to treatment and may even prove useful in diagnosis. Future work using more sensitive targeted proteomic approaches to investigate levels of specific cytokines could be useful. Since finding differences between FFA and lichen planopilaris promises to help elucidate the pathophysiological basis for these conditions [23], a comparison of their protein profiles in scalp and other sites could be enlightening. Such an investigation would be a useful adjunct to studies of efficacy of lichen planopilaris treatment with peroxisome proliferator associated receptor agonists, which have shown clinical promise in some cases [24].

## Conclusion

FFA appears to be an inflammatory syndrome of autoimmune origin that targets hair follicles, resulting in hair loss. Whether the epidermis is affected by this condition was not certain previously. This study sought evidence that protein expression in the interfollicular epidermis of the scalp and forehead is altered. Using non-invasive collection of corneocytes from the epidermal surface (stratum corneum), proteomic analysis gave clear evidence that the expression levels of numerous corneocyte proteins were perturbed in this syndrome. By contrast, epidermal protein profiles from subjects exhibiting AGA differed little from those in asymptomatic subjects. This work provides a basis for quantitative measures of departure from normal in frontal fibrosing alopecia by assaying forehead epidermis. This approach may be useful in monitoring response to treatment and avoids invasive biopsy.

## Supporting information

S1 Fig Ingenuity pathway analysis. Submission of data from the comparison of FFA with control forehead profiles resulted in identification of two pathways that appeared suppressed, both containing PLCD1 and three 14-3-3 adaptor proteins (SFN, YWHAZ, YWHAE).
(PDF)

S2 Fig Comparison of expression levels in forehead proteins in frontal fibrosing alopecia (FFA), androgenetic alopecia (AGA) and control (CON) samples. Relative expression levels were calculated from log FC values in S3 Table. Proteins are identified by gene names (abbreviated) to avoid ambiguity. (A) Expressed at higher levels in FFA samples are (1) GAPDH, (2) SERPINB12, (3) KRT77, (4) ALOXE3, (5) CAT, (6) KRT19, (7) TXN, (8) BLMH (9) KRT4, (10) GSDMA, (11) PRDX4, (12) HSPD1, (13) CAPNS2, (14) GDPD3, (15) LGALSL, (16) SERPINB7, (17) GDA, (18) PEBP1, (19) PSMB7, (20) RO60, (21) STS, (22) IDH1, (23) PSMB5, (24) HAL and (25) PLCXD1. (B) Expressed at lower levels in FFA samples are (1) KRTAP9-3, (2) KRTAP9-9, (3) KRTAP2-3, (4) DSG4, (5) KRT38, (6) KRT84, (7) LCE2C, (8) LCE2B, (9) KRT39, (10) LRRC15, (11) LCE1A, (12) KRTAP13-2, (13) LGALS3, (14) KRT82, (15) VSIG8, (16) KRT40, (17) KRTAP10-10, (18) KRTAP3-1, (19) KRTAP16-1, (20) TUBB4B, (21) H1-4, (22) KRT35, (23) S100A8, (24) S100A3, (25) KRT31, (26) LCE1F, (27) KRT86, (28) KRT32, (29) KRT36, (30) S100A2, (31) KRT75, (32) KRT85, (33) PRDX6, (34) HSPA2, (35) CALML3, (36) PLCD1, (37) YWHAZ, (38) SFN, (39) HSPA8, (40) YWHAE, (41) S100A7, (42) LMNA, (43) SPRR2G, (44) SERPINB3, (45) S100A9, (46) KRT16 and (47) PKP1.
(PDF)

S1 Table Weighted spectral counts in samples collected from FFA, AGA or control (CON) samples. (XLSX)

S2 Table Estimates of relative protein amounts in samples using label-free quantitation. (XLSX)

S3 Table Two-way statistical comparisons using the weighted spectral counts from S2 Table. (XLSX)
