# Supplementary material for: Protein profiling of forehead epidermal corneocytes distinguishes frontal fibrosing from androgenetic alopecia
Source: PLoS One. 2023 Mar 31;18(3):e0283619. doi: 10.1371/journal.pone.0283619 (PMC10065298; doi:10.1371/journal.pone.0283619)
Supplement: S1 Fig — Submission of data from the comparison of FFA with control forehead profiles resulted in identification of two pathways that appeared suppressed, both containing PLCD1 and three 14-3-3 adaptor proteins (SFN, YWHAZ, YWHAE). (PDF) [file pone.0283619.s001.pdf]

6 molecule(s) associated with **Protein Kinase A Signaling** at **Observation 4** [Ratio: 6/407 (0.015)] [z-score: -2.236] [p-value: 6.17E-04]

Add To My Pathway | Add To My List | Create Dataset | Customize Table | Expand

Symbol	Entrez Gene ...	Identifier	Measurement	+ Add/Remove column(s)		Expected
		Entrez Gene/...	Expr Log Ratio	Expr p-value	Expr False Di...	
**GDPD3**	glycerophosphodies	**GDPD3**	↑1.637	6.88E-03	3.08E-02	
**H1-4**	H1.4 linker histone,	**H1-4**	↓-2.426	1.22E-04	1.41E-03	↑ Up
**PLCD1**	phospholipase C de	**PLCD1**	↓-1.707	8.70E-03	3.60E-02	↑ Up
**SFN**	stratifin	**SFN**	↓-1.586	1.12E-02	4.30E-02	↑ Up
**YWHAE**	tyrosine 3-monooxy	**YWHAE**	↓-1.487	7.07E-03	3.08E-02	↑ Up
**YWHAZ**	tyrosine 3-monooxy	**YWHAZ**	↓-1.649	7.00E-03	3.08E-02	↑ Up

5 molecule(s) associated with **14-3-3-mediated Signaling** at **Observation 4** [Ratio: 5/127 (0.039)] [z-score: -2] [p-value: 1.90E-05]

Add To My Pathway | Add To My List | Create Dataset | Customize Table | Expand

Symbol	Entrez Gene ...	Identifier	Measurement	+ Add/Remove column(s)		Expected
		Entrez Gene/...	Expr Log Ratio	Expr p-value	Expr False Di...	
**PLCD1**	phospholipase C de	**PLCD1**	↓-1.707	8.70E-03	3.60E-02	↑ Up
**SFN**	stratifin	**SFN**	↓-1.586	1.12E-02	4.30E-02	↑ Up
**TUBB4B**	tubulin beta 4B clas	**TUBB4B**	↓-2.455	7.02E-04	5.56E-03	
**YWHAE**	tyrosine 3-monooxy	**YWHAE**	↓-1.487	7.07E-03	3.08E-02	↑ Up
**YWHAZ**	tyrosine 3-monooxy	**YWHAZ**	↓-1.649	7.00E-03	3.08E-02	↑ Up

S1 Figure. Ingenuity Pathway Analysis. Submission of data from the comparison of FFA with control forehead profiles resulted in identification of two pathways that appeared suppressed, both containing PLCD1 and three 14-3-3 adaptor proteins (SFN, YWHAZ, YWHAE).
