# Supplementary material for: Protein profiling of forehead epidermal corneocytes distinguishes frontal fibrosing from androgenetic alopecia
Source: PLoS One. 2023 Mar 31;18(3):e0283619. doi: 10.1371/journal.pone.0283619 (PMC10065298; doi:10.1371/journal.pone.0283619)
Supplement: S2 Fig — Relative expression levels were calculated from log FC values in S3 Table. Proteins are identified by gene names (abbreviated) to avoid ambiguity. (A) Expressed at higher levels in FFA samples are (1) GAPDH, (2) SERPINB12, (3) KRT77, (4) ALOXE3, (5) CAT, (6) KRT19, (7) TXN, (8) BLMH (9) KRT4, (10) GSDMA, (11) PRDX4, (12) HSPD1, (13) CAPNS2, (14) GDPD3, (15) LGALSL, (16) SERPINB7, (17) GDA, (18) PEBP1, (19) PSMB7, (20) RO60, (21) STS, (22) IDH1, (23) PSMB5, (24) HAL and (25) PLCXD1. (B) Expressed at lower levels in FFA samples are (1) KRTAP9-3, (2) KRTAP9-9, (3) KRTAP2-3, (4) DSG4, (5) KRT38, (6) KRT84, (7) LCE2C, (8) LCE2B, (9) KRT39, (10) LRRC15, (11) LCE1A, (12) KRTAP13-2, (13) LGALS3, (14) KRT82, (15) VSIG8, (16) KRT40, (17) KRTAP10-10, (18) KRTAP3-1, (19) KRTAP16-1, (20) TUBB4B, (21) H1-4, (22) KRT35, (23) S100A8, (24) S100A3, (25) KRT31, (26) LCE1F, (27) KRT86, (28) KRT32, (29) KRT36, (30) S100A2, (31) KRT75, (32) KRT85, (33) PRDX6, (34) HSPA2, (35) CALML3, (36) PLCD1, (37) YWHAZ, (38) SFN, (39) HSPA8, (40) YWHAE, (41) S100A7, (42) LMNA, (43) SPRR2G, (44) SERPINB3, (45) S100A9, (46) KRT16 and (47) PKP1. (PDF) [file pone.0283619.s002.pdf]

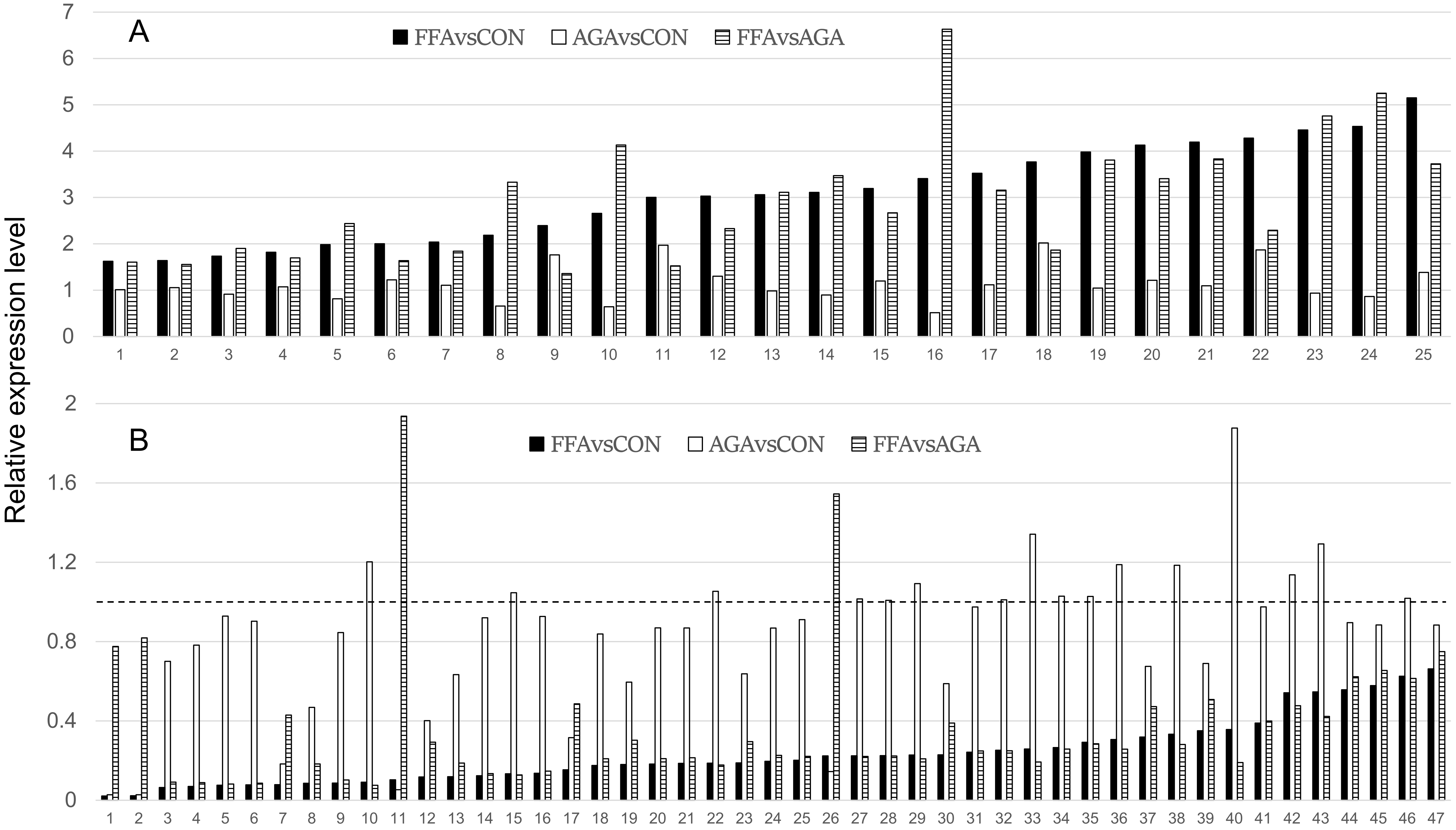

A
B
FFAvsCON
AGAvsCON
FFAvsAGA
Relative expression level

S2 Figure. Comparison of expression levels in forehead proteins in frontal fibrosing alopecia (FFA), androgenetic alopecia (AGA) and control (CON) samples. Relative expression levels were calculated from log FC values in S4 Table. Proteins are identified by gene names (abbreviated) to avoid ambiguity. (A) Expressed at higher levels in FFA samples are (1) GAPDH, (2) SERPINB12, (3) KRT77, (4) ALOXE3, (5) CAT, (6) KRT19, (7) TXN, (8) BLMH (9) KRT4, (10) GSDMA, (11) PRDX4, (12) HSPD1, (13) CAPNS2, (14) GDPD3, (15) LGALSL, (16) SERPINB7, (17) GDA, (18) PEBP1, (19) PSMB7, (20) RO60, (21) STS, (22) IDH1, (23) PSMB5, (24) HAL and (25) PLCXD1. (B) Expressed at lower levels in FFA samples are (1) KRTAP9-3, (2) KRTAP9-9, (3) KRTAP2-3, (4) DSG4, (5) KRT38, (6) KRT84, (7) LCE2C, (8) LCE2B, (9) KRT39, (10) LRRC15, (11) LCE1A, (12) KRTAP13-2, (13) LGALS3, (14) KRT82, (15) VSIG8, (16) KRT40, (17) KRTAP10-10, (18) KRTAP3-1, (19) KRTAP16-1, (20) TUBB4B, (21) H1-4, (22) KRT35, (23) S100A8, (24) S100A3, (25) KRT31, (26) LCE1F, (27) KRT86, (28) KRT32, (29) KRT36, (30) S100A2, (31) KRT75, (32) KRT85, (33) PRDX6, (34) HSPA2, (35) CALML3, (36) PLCD1, (37) YWHAZ, (38) SFN, (39) HSPA8, (40) YWHAE, (41) S100A7, (42) LMNA, (43) SPRR2G, (44) SERPINB3, (45) S100A9, (46) KRT16 and (47) PKP1.
